# Modulating Sterol Concentrations in Infant Formula Influences Cholesterol Absorption and Synthesis in the Neonatal Piglet

**DOI:** 10.3390/nu10121848

**Published:** 2018-12-01

**Authors:** Elizabeth A Babawale, Peter JH Jones, Kelly E Mercer, Haixia Lin, Laxmi Yeruva, Fabiana Bar Yoseph, Shane M Rutherfurd

**Affiliations:** 1 Department of Food and Human Nutritional Science, University of Manitoba, Winnipeg, MB R3T 2N2, Canada; abosedee@myumanitoba.ca; 2 Richardson Centre for Functional Foods and Nutraceuticals, University of Manitoba, Winnipeg, MB R3T 6C5, Canada; 3 Arkansas Children’s Nutrition Center, Little Rock, AR 72202, USA; kmercer@uams.edu (K.E.M.); HLin@uams.edu (H.L.); VLYeruva@uams.edu (L.Y.); 4 Department of Pediatrics, University of Arkansas for Medical Sciences, Little Rock, AR 72202, USA; 5 Arkansas Children’s Research Institute, Little Rock, AR 72202, USA; 6 Clinical Development, Enzymotec Ltd., Kfar Baruch 23106, Israel; 7 Riddet Institute, Massey University, Palmerston North 4442, New Zealand; s.m.rutherfurd@kinect.co.nz

**Keywords:** cholesterol, plant sterols, infant formula, synthesis, absorption

## Abstract

Formula-fed infants present higher cholesterol synthesis rates and lower circulating cholesterol during the postnatal feeding period compared to breast-fed infants, though the mechanisms underlying this phenotype are not fully understood. Typical infant formulas contain vegetable-based fats, inherently including phytosterols (PS), which are structurally similar to cholesterol and may interfere with their absorption. A seven-day old piglets model was used to test the inhibitory effects of PS on cholesterol absorption during postnatal feeding. Following feeding for 21 days with milk-based formulas containing PS and cholesterol levels resembling those in formulas or human-milk, apparent cholesterol digestibility was analyzed in ileal digesta, and cholesterol, PS, and cholesterol synthesis markers were analyzed in plasma and liver samples. Ileal cholesterol digestibility content was increased in the piglets fed low PS formulas and the rate of the hepatic cholesterol synthesis, as determined by the lathosterol-to-cholesterol ratios (L:C), was decreased in the piglets fed LP-formulas and corresponded to reduced nuclear expression of SREBP2 relative to those fed HP-formulas. These results are consistent with the hypothesis that PS in formula can inhibit cholesterol absorption and enhance cholesterol synthesis. Whether or not this leads to entrainment of cholesterol synthesis later in life via early programming awaits further research.

## 1. Introduction

Cholesterol is essential for life, especially during the rapid growth of infants, as it is a structural element of the cell membrane lipid layer; a substrate for the production of steroid hormones, vitamin D, and bile acids [1]; crucial for proper brain development and myelin formation; and plays a key role in lipoprotein synthesis and metabolism [2]. Moreover, studies suggest an influence of early nutrition on blood cholesterol levels and thus cardiovascular disease risk (CVD) during adulthood [3,4,5].

While human milk is a rich source of cholesterol, containing 90 to 150 mg/L cholesterol [6], most infant formulas are vegetable oils based, containing a significantly lower level of cholesterol (15–50 mg/L), which originates mainly from dairy milk fat [2,7]. This difference in cholesterol content results in about a three to five times higher intake of cholesterol in breast-fed infants compared to formula-fed infants [2,8]. Interestingly, formula-fed infants have increased fractional cholesterol synthesis rates relative to those infants that are breast-fed, suggesting endogenous cholesterol synthesis as a reciprocal mechanism in response to the low dietary uptake [9,10,11,12].

Vegetable oils-based formulas not only provide low cholesterol levels, but they inherently contain plant sterols, called phytosterols (PS), such as Campsterol, Brassicasterol, Stigmasterol, and Sitosterol [13]. PS are known to inhibit cholesterol absorption by competing in micelle formation, and are substrates for the intestinal sterol transporter Niemann-Pick C1-Like 1 (Npc1l1), which mediates the extracellular cholesterol transport across the brush border membrane in the ileum [14,15]. However, Npc1l1 preferentially absorbs cholesterol [16,17]. In adults, PS are barely absorbed by the intestine (about 5%, for review see [14]), but little is known about the importance of PS in infants’ diets. Given that plasma cholesterol concentrations are determined by the balance between dietary sterol absorption from the gastrointestinal tract and whole body endogenous cholesterol synthesis [18], it is possible that circulating PS concentrations during infancy could be important for proper lipid programming and metabolism in later life [19]. However, the long-term effects of consuming diets relatively high in PS during infancy are yet to be explored [6].

Although efforts have been made to mimic human breast milk composition by the supplementation of infant formula with different cholesterol sources [19], the potential relationship between dietary PS and cholesterol concentrations with regards to cholesterol absorption from infant formulas has not been addressed. We hypothesized that a low PS modified vegetable oil should facilitate increased cholesterol absorption and lead to lower endogenous cholesterol synthesis. Therefore, the objective of this study was to investigate the effect of infant formulas containing different levels of PS and cholesterol on circulating cholesterol concentrations, apparent ileal cholesterol digestibility, and endogenous cholesterol synthesis by using a neonatal piglet as a model.

## 2. Materials and Methods

### 2.1. Animals Experiments

The impact of dietary PS and cholesterol concentration on cholesterol absorption and endogenous cholesterol synthesis was determined in neonatal piglets given one of four dietary treatments, following approval by Massey University’s Ethics Committee. Thirty-two seven-day old male piglets were housed in purpose-built plastic metabolism crates in a temperature-controlled room maintained at 28 ± 2 °C with a 16:8 h light: dark cycle. Piglets were initially weighed and randomly allocated to one of the four experimental diets such that there were eight piglets per treatment. Piglets were fed 345 g of prepared liquid formula per kg of body weight per day [20]. The piglets were trained to drink using a bottle and teat and were acclimatized to their environment and diet over the first six days of the study, during which time they were fed their daily ration over 17 meals given hourly from 06:00 h to 10:00 h. For the remainder of the experimental period (16 days), the piglets received their daily food rations as seven meals given every 2.5 h from 06:30 h to 21:30 h. From day 14 to 21, titanium dioxide (an indigestible marker) was also added to the prepared formula at a concentration of 3 g per kg of dry matter. The daily formula ration was readjusted weekly based on the body weights of the piglets. Any formula that was not consumed was collected, dried, and weighed for each piglet in order to determine dietary intake. The dietary treatments (Table 1), prepared at the Food Pilot Plant (Massey University), contained different combinations of PS and cholesterol concentrations commensurate with those in human milk or standard vegetable oil-based infant formulas. 

On day 22 of the study, piglets were fed their respective formula at hourly intervals starting at 06:30 h. Seven hours after the start of feeding, each piglet was anaesthetised using a cocktail of Xylazine, Zolazepam, and Tiletamine. While under anaesthesia, blood was collected via cardia puncture and aliquoted into EDTA-treated vacutainers. Then, piglets were euthanized using a lethal cardiac injection of sodium pentobarbitone, after which liver tissue and ileal digesta were collected and stored at −80 °C for analysis. Circulating total-cholesterol and LDL-cholesterol levels were measured in the plasma of all diet groups using commercially available assays and a Vitros 350 autoanalyzer. 

### 2.2. Determination of Cholesterol and Other Sterols in Plasma, Ileal Digesta and Liver Tissues

All standards, potassium hydroxide (KOH) salt, and the internal standard were obtained from Sigma Aldrich Canada Ltd (Oakville, ON, Canada). Solvents were obtained from Fischer Scientific. Sample extraction was performed according to Jones et al. [21], with a few modifications. Sterols were extracted twice and dried under N_2_ before being saponified with methanol-KOH, using 5α-cholestane as an internal standard. The dried residue was re-suspended in hexane and 100 µL of HMDS + TMCS + Pyridine (3:1:9) was added for derivatization. Extracted samples were then transferred to Gas chromatography (GC) vials for analysis using a GC with Flame Ionization Detector (GC-FID) with an SAC-5 fused silica capillary column with a 30 m × 0.25 mm × 0.25 µm film thickness. An initial run temperature of 130 °C was used for 2 min, before increasing the temperature to 270 °C at 30 °C per min, holding it at 270 °C for 10 min, increasing the temperature to 290 °C at 10 °C per min, holding it for 9 min, and then finally ramping it up to 320 °C at 40 °C per min and holding it for 5 min. Helium was used as the carrier gas at a flow rate of 1.0 mL per min. Injector and detector temperatures were 280 °C and 300 °C, respectively. Sterols were identified by comparing the retention time of the peaks in the sample with the retention time of known sterols (cholesterol, campesterol, sitosterol, sitostanol, lathosterol, and desmosterol) in an external standard. 

### 2.3. Determining the Titanium Dioxide Concentration in Diets and Ileal Digesta 

The titanium content of the diets and digesta were determined based on the method of Short et al. [22]. Briefly, samples were ashed before being digested in 60% (*v*/*v*) sulphuric acid. The mixture was then incubated with 30% H_2_O_2_ and the absorbance read at 405 nm. Cholesterol flow rate at the terminal ileum was calculated as follows (units are mg·kg^−1^ DM (dry matter)): Ileal cholesterol flow (mg·kg^−1^ dry matter intake (DMI)) = Digesta cholesterol × Formula titanium dioxide/Digesta titanium dioxide. The apparent ileal cholesterol digestibility value was calculated using the following equation (units are mg/kg DMI): Apparent ileal cholesterol digestibility (%) = (Formula cholesterol − Ileal cholesterol flow)/Formula cholesterol × 100.

### 2.4. Determination of Cholesterol in Ileum 

The total cholesterol, free cholesterol, and cholesteryl esters were extracted from the ileum (100 mg) by chloroform/isopropanol/NP-40 (7:11:0.1) and concentrations were determined using a commercially available fluorometric cholesterol/cholesteryl ester quantitative assay (Abcam, Ab65359) as per the manufacturer’s instructions.

### 2.5. Quantitative Real-Time RT-PCR 

Total RNA was extracted from 50–100 mg of sample tissue, i.e., liver and ileum, using the miRNeasy Mini kit (Qiagen, Valencia, CA, USA), in accordance with the manufacture’s protocol. A total of 1 µg of RNA from each sample was reverse transcribed in a 20 µL reaction using the TaqMan High Capacity cDNA Reverse Transcription Kit (Life Technologies, Foster City, CA, USA), following the manufacturer’s instruction. Before cDNA synthesis, the RNA concentration and integrity were measured in the LVis Plate using a microplate reader (BMG LABTECH, Cary, NC, USA) and the Experion RNA StdSens Analysis Kit (Bio-Rad, Hercules, CA, USA). RNA with an integrity score >7.5 integrity was used for cDNA synthesis. cDNA was amplified using Fast Sybr Green Master Mix, according to the manufacturer’s protocol. The 15 µL reaction was conducted on a 7500 Fast Applied Biosystems Real-Time PCR System (Life Technologies, Foster City, CA, USA). Sample cycle threshold (Ct) values for each sample were determined using the Applied Biosystems software. Relative gene expression was determined by calculating the 2^−∆Ct^ method relative to *B_2_M* gene amplification (*B_2_M*, Genebank NM_213978, 70 bp primers, Qiagen, Valencia, CA USA). Primer sequences used have been previously published [23].

### 2.6. Western Blot Analysis

Nuclear and membrane protein fractions were obtained using NE-PER Nuclear and Cytoplasmic Extraction Reagents (Thermo Scientific, Waltham, MA, USA), following the manufacturers’ instructions. Gel electrophoresis was performed, and proteins were transferred to a PVDF membrane. Membranes were first incubated for 1 h in Tris-buffered saline (TBS) containing 0.05% Tween-20 and 5% non-fat dry milk to reduce non-specific antibody binding, and then incubated overnight at 4 °C with either anti-SREBP2 (1:5000) or anti-LDLR (1:5000) rabbit polyclonal antibodies from Lifespan Biosciences (Seattle, WA, USA), diluted in TBS containing 0.1% Tween-20 and 5% Bovine serum albumin (BSA). After incubation with horseradish peroxidase-conjugated secondary antibodies (1:10,000 mouse anti-rabbit; Santa Cruz Technology, Santa Cruz, CA, USA), membranes were covered in ECL Plus Western Blotting Detection Reagent (Thermo Scientific) and developed and imaged using an Amersham® Imager 600 (GE Healthcare, Hammersmith, UK). The density of protein bands was determined using Quantity One software (Bio-Rad, Hercules, CA, USA) and expressed relative to total protein.

### 2.7. Statistical Analysis

Results are presented as means and standard error of the means. For most analyses the effect of PS, cholesterol, and the interaction of these two components were determined by two-way ANOVA followed by Student Newman-Keuls post hoc analysis. Other analyses, i.e., food intake, weight gain, and cholesterol digestibility, were analyzed statistically by one-way ANOVA using GLM procedures (PROC UNIVARIATE (SAS, 2009) SAS, 2009, Cary, NC, USA). Where statistically significant (*p* < 0.05) effects were observed, individual means were compared using the Tukey test. 

## 3. Results

### 3.1. Food Intake and Body Weight of Piglets Receiving Different Infant Formulas

Male, seven-day old piglets (*n* = 8 per diet group) were fed different infant formulas containing high or low PS concentrations (HP and LP, respectively), plus or minus added cholesterol (FC and F respectively) for 16 days. During this study, piglets readily adapted to bottle feeding, gained weight, and remained healthy throughout the trial. There was no occurrence of diarrhoea in any of the piglets throughout the study. The percentage of the weekly ration consumed and body weights of the piglets across treatment groups are presented in Table 2. No statistically significant differences were observed for either percentage of the weekly ration consumed or piglet’s body weight across treatment groups throughout the trial.

### 3.2. Plasma and Liver PS Levels

**Figure 1:**
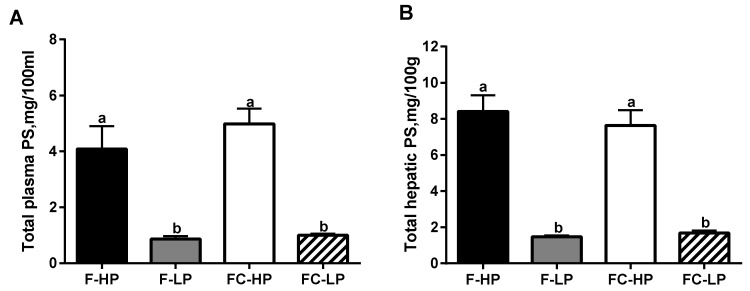
Total PS concentrations in plasma (**A**) and liver (**B**) of piglets fed different infant formulas containing high (F-HP) or low (F-LP) PS concentrations, supplemented with cholesterol (FC-HP, FC-LP) to achieve concentrations similar to human milk. Data are means ± Standard error of the mean (SEM), *n* = 8/diet group. Statistical differences were determined by two-way ANOVA; for total plasma PS, *p* < 0.001 for PS, *p* = 0.360 for cholesterol, and *p* = 0.427 for interaction; for total hepatic PS, *p* < 0.001 for PS, *p* = 0.305 for cholesterol, and *p* = 0.407 for interaction; Student Newman-Keuls post hoc analysis. Bars without a common letter significantly differ from each other, *p* < 0.05.

In all diet groups, individual PS (campesterol, sitosterol, and sitostanol) were detected in plasma and liver samples (Supplementary Table S1). Total PS concentrations in the plasma and liver samples are shown in Figure 1. Piglets fed the low PS formulas (F-LP and FC-LP) exhibited significantly lower PS concentrations in plasma and liver samples compared to the high PS formulas (F-HP and FC-HP formulas). 

### 3.3. Plasma Total-Cholesterol and LDL-Cholesterol Levels

Total-cholesterol concentrations were 2.71 ± 0.18, 2.70 ± 0.08, 3.00 ± 0.07, and 3.05 ± 0.09 mM for F-HP, F-LP, FC-HP, and FC-LP fed piglets, respectively; no statistical differences were observed (two-way ANOVA followed by Student Newman-Keuls post hoc analysis, *p* = 0.899 for PS, *p* = 0.021, for cholesterol, *p* = 0.774, interaction). However, we did observe an increase in plasma LDL-cholesterol in the cholesterol supplemented groups (0.98 ± 0.07 and 1.01 ± 0.05 mM) for FC-HP and FC-LP fed piglets, respectively, relative to the F-HP and F-LP fed piglets (0.71 ± 0.08 and 0.71 ±0.09 mM), respectively (two-way ANOVA, followed by Student Newman-Keuls post hoc analysis, *p* = 0.858 for PS, *p* = 0.001 for cholesterol, *p* = 0.896, interaction).

### 3.4. Apparent Ileal Cholesterol Digestibility and Apparent Ileal Digestible Cholesterol Content 

The apparent ileal digestibility of cholesterol was determined by comparing the sterol concentration in the formula with the unabsorbed cholesterol present at the end of the small intestine. The apparent ileal cholesterol digestibility and the apparent ileal digested cholesterol content of the four test formulas determined in the neonatal piglet are shown in Table 3. Relative to the control diet (F-HP), the apparent ileal cholesterol digestibility was significantly higher (≥2-fold) in the other diet groups. As expected, the apparent ileal digestible cholesterol content in the cholesterol supplemented diets (FC-HP, FC-LP) was 3.9- to 10-fold higher relative to F-HP and F-LP diets, *p* < 0.01. Interestingly, we did observe a two-fold increase in apparent ileal digestible cholesterol content in the F-LP diet relative to the F-HP, yet this difference was not significant, *p* > 0.05. 

### 3.5. Cholesterol Synthesis and Transport in the Ileum

**Figure 2:**
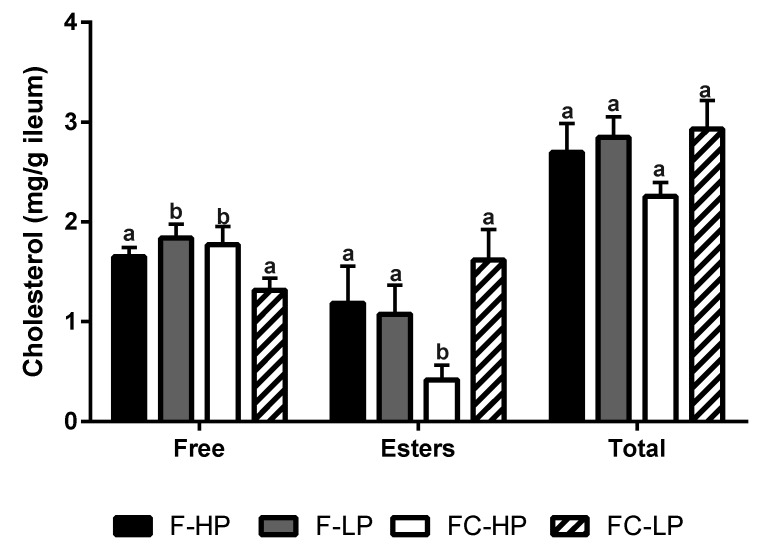
Free cholesterol, cholesterol esters, and total cholesterol (free + esters) concentrations in the ileal tissue of piglets fed different infant formulas containing high (F-HP) or low (F-LP) PS concentrations, supplemented with cholesterol (FC-HP, FC-LP) to achieve concentrations similar to human milk. Data are means ± SEM, *n* = 8/diet group. Statistical differences were determined by Two-way ANOVA; for free cholesterol, *p* = 0.371 for PS, *p* = 0.183 for cholesterol, and *p* < 0.05 for interaction; for cholesterol esters, *p* = 0.074 for PS, *p* = 0.701 for cholesterol, and *p* < 0.05 for interaction; for total cholesterol, *p* = 0.470 for PS, *p* = 0.098 for cholesterol, and *p* = 0.289 for interaction, Student Newman-Keuls post hoc analysis. Bars without a common letter significantly differ from each other, *p* < 0.05.

As shown in Figure 2, cholesterol concentrations were measured in the ileal tissue of piglets fed infant formulas containing different PS and cholesterol concentrations. In comparison to the high PS low cholesterol control group (F-HP), ileal free cholesterol concentrations were slightly increased by 7% and 12% in the F-LP and FC-HP fed groups (*p* < 0.05). More importantly, free cholesterol concentrations were significantly lower in the FC-LP fed group compared to the F-HP, F-LP, and FC-HP fed groups by 25%, 39%, and 34%, respectively. Surprisingly, in the FC-HP fed group, cholesterol esters were significantly lower in comparison to the F-HP, F-LP, and FC-LP fed piglets, with the greatest decrease observed in the FC-HP compared to the FC-LP fed group. Overall, we observed a trend for decreased total cholesterol concentrations in the FC-HP fed group relative to the F-LP group and F-HP control (*p* = 0.071). We did observe a significant difference in total cholesterol content between FC-HP vs. FC-LP (Figure 2).

**Figure 3:**
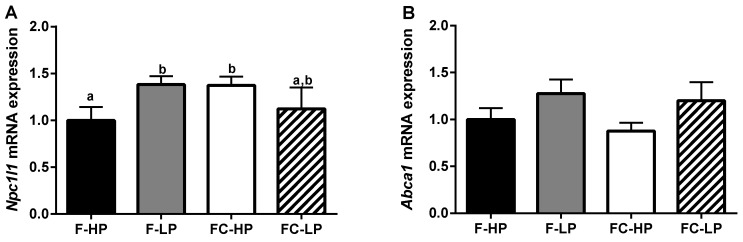
*Npc1l1* (**A**) and *Abca1* (**B**) mRNA fold change in ileal tissue of piglets fed different infant formulas containing high (F-HP) or low (F-LP) PS concentrations, supplemented with cholesterol (FC-HP, FC-LP) to achieve concentrations similar to human breast milk. Data are means ± SEM, *n* = 8/diet group. Statistical differences were determined by two-way ANOVA; for *Npc1l1* mRNA, *p* = 0.653 for PS, *p* = 0.693 for cholesterol, and *p* = 0.037 for interaction; for *Abca1* mRNA, *p* = 0.058 for PS, *p* = 0.512 for cholesterol, and *p* = 0.407 for interaction; Student Newman-Keuls post hoc analysis. Bars without a common letter significantly differ from each other, *p* < 0.05.

In the ileum, mRNA expression of transporters involved in intestinal cholesterol uptake (Niemann-Pick C1-like 1, *Npc1l1*) and efflux (Adenosine Triphosphate (ATP) binding cassette subfamily A, member 1, *Abca1*; ATP binding cassette subfamily G, members 5 and 8, *Abcg5/8*) were also measured by real-time RT-PCR. For *Npc1l1*, mRNA expression increased in the F-LP and FC-HP groups relative to the F-HP control group (Figure 3A). *Npc1l1* gene expression was decreased (20%) in the FC-LP group, relative to the FC-HP group, yet this difference was not significant (*p* < 0.100). For *Abca1*, we observed a trend for increased mRNA expression in the low PS diet groups (F-LP, FC-LP) relative to the high PS groups, F-HP and FC-HP, *p* = 0.058 (Figure 3B). Gene expression of *Abcg5/8* did not differ between diet groups (data not shown).

### 3.6. Hepatic Cholesterol Synthesis

Concentrations of cholesterol synthesis precursors present in the plasma and liver of piglets receiving the four test formulas are shown in Table 4. Interestingly, the desmosterol-to-cholesterol ratio (D:C ratio) and lathosterol-to-cholesterol ratio (L:C ratio) were significantly lower in the FC-LP group, suggesting reduced cholesterol synthesis rates in this diet group relative to the other diets. In the liver, the D:C ratios were lower in both the F-LP and FC-LP groups, relative to the high PS formula fed groups, F-HP and FC-HP. In contrast, the L:C ratios were significantly lower in the cholesterol supplemented groups (FC-HP, FC-LP) relative to the non-supplemented formulas (F-HP, F-LP), with a trend for the lowest L:C ratio in the FC-LP group relative to the FC-HP group, *p* = 0.062 (Table 4). 

**Figure 4:**
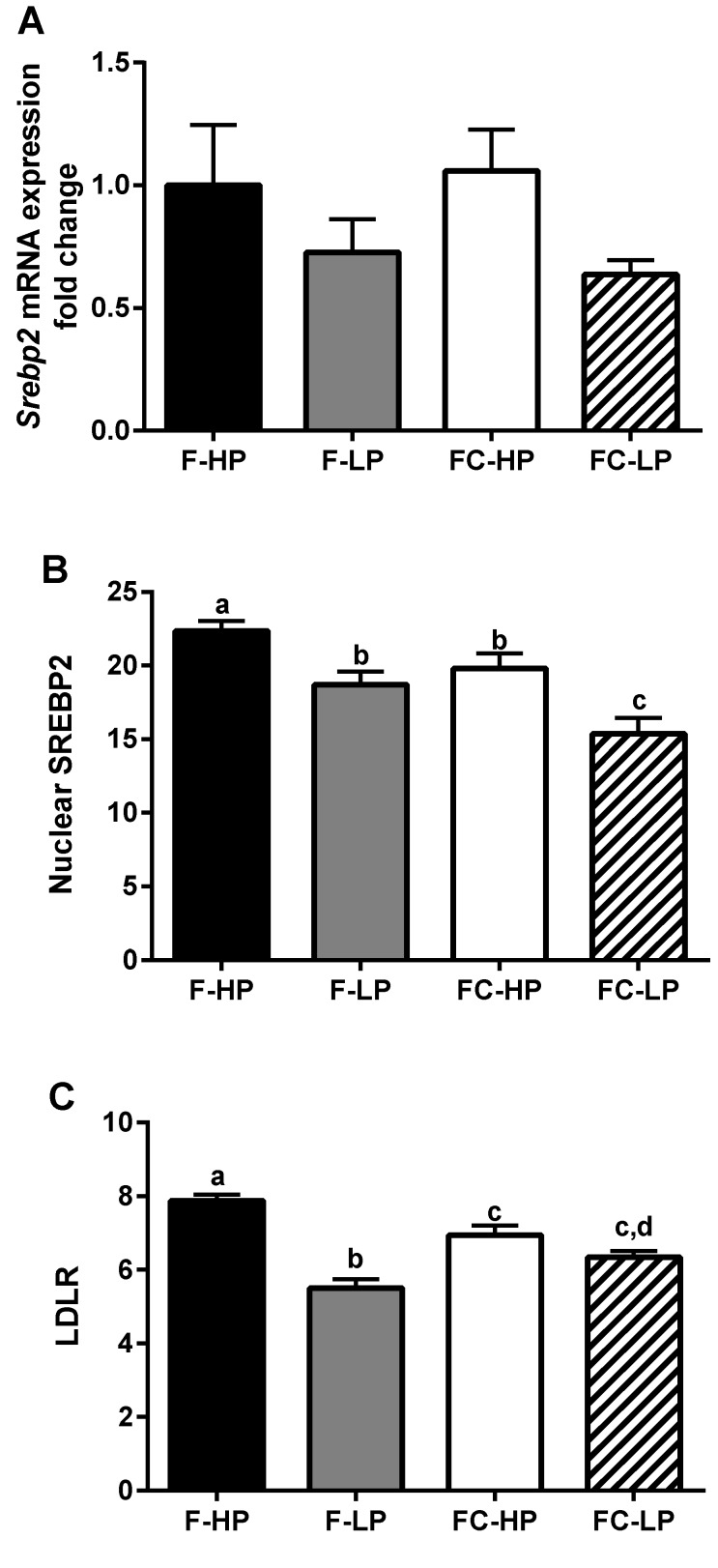
*Srebp2* mRNA fold change (**A**) and nuclear protein expression (**B**), and LDLR protein expression (**C**) in the liver of piglets fed different infant formulas containing high (F-HP) or low (F-LP) PS concentrations, supplemented with cholesterol (FC-HP, FC-LP) to achieve concentrations similar to human breast milk. Data are means ± SEM, *n* = 8/diet group. Statistical differences were determined by two-way ANOVA; for *Srebp2* mRNA, *p* = 0.056 for PS, *p* = 0.833 for cholesterol, and *p* = 0.427 for interaction; for SREBP2, *p* < 0.001 for PS, *p* = 0.004 for cholesterol, and *p* = 0.673 for interaction; for LDLR, *p* < 0.001 for PS, *p* = 0.881 for cholesterol, and *p* < 0.001 for interaction. Student Newman-Keuls post hoc analysis. Bars without a common letter significantly differ from each other, *p* < 0.05.

These findings suggest that hepatic cholesterol synthesis is reduced in piglets fed low PS formula supplemented with cholesterol, relative to the other diet groups. Consistent with these findings, we observed a trend for decreased mRNA expression (~35%) of the transcription factor controlling hepatic cholesterol synthesis, sterol regulatory element-binding protein-2 (*Srebp2*) in the low PS groups, *p* = 0.056, (Figure 4A); nuclear SREBP2 expression was also significantly lower in the low PS groups (F-LP, FC-HP) relative to the high PS formula fed groups (F-HP, FC-HP), with the lowest expression observed in the FC-LP fed piglets relative to all other diet groups (Figure 4B). The membrane expression of the low density lipoprotein receptor (LDLR), a target for SREBP2 regulation, was also decreased in groups receiving the low PS formulas (F-LP, FC-LP) relative to the high PS formulas (Figure 4C).

## 4. Discussion

Breast-fed infants consuming up to five-fold greater amounts of cholesterol than formula-fed infants have been found to be less prone to obesity and related diseases, such as cardiovascular diseases, during adulthood; the latter has been hypothesized to be attributed—at least in part—to exposure to dietary cholesterol early in life. Also, evidence from epidemiological studies associates initial breastfeeding, especially exclusive breastfeeding, with lower blood cholesterol levels during adulthood [24]. As such, the importance of providing the growing infant with adequate dietary cholesterol cannot be overemphasized. Several studies report different approaches to enhancing circulating cholesterol in infants consuming infant formula, by using various modifications, including blending different oil sources or the addition of cholesterol in modified infant formula, all in an attempt to increase the cholesterol concentrations in infant formula [1,10,25,26,27,28,29]. The use of PS-reduced vegetable oils in an attempt to improve cholesterol absorption, in addition to cholesterol supplementation, has not been previously investigated in infant formula. Therefore, we utilized a neonatal piglet model of postnatal feeding to test the hypothesis that PS impede cholesterol uptake, resulting in increased hepatic cholesterol synthesis rates.

As expected, piglets fed formulas with a lower PS concentration exhibited less PS in plasma, as well as in the liver tissue. These results are in line with Mellies et al. [6], who showed a direct correlation between the PS concentrations in the human milk and in the plasma of breastfed infants. In the present work, the reduction in PS intake led to an increase in the absorption of cholesterol, as revealed by the higher cholesterol digestibility in piglets fed the formulas with the reduced PS. The enhancement of cholesterol absorption provides higher cholesterol availability from the diet and enables lower cholesterol endogenous synthesis. 

Apparent ileal cholesterol digestibility ranged from 32% to 85% across the test animals in this study. While there is a dearth of information relating to the cholesterol digestibility of formulas in infants, the latter values were similar to those reported for the cholesterol absorption of mixed diets when determined in adult humans using techniques such as the dual isotope label method [30,31,32,33]. Though the results are in line with the reported range, there was a significant difference observed between the regular infant formula diet and the other formulas that differed in PS levels or cholesterol level; however, digestibility was highest for the formula with the high cholesterol and the low PS levels. The cholesterol digestibility was two times higher by just reducing the diet PS; and up to 10 times higher by reducing the dietary PS and supplementing the formula with cholesterol. Consistent with these findings, there were higher cholesterol ester concentrations (3-fold) in the ileum of piglets fed the low PS, high cholesterol formula (FC-LP), relative to the high PS, high cholesterol formula (FC-HP). In the ileum, the fate of absorbed cholesterol is dependent on its physicochemical state, whether it is esterified via acylCoA:cholesterol acyltransferase 2 (*Acat2*) or remains in the cellular and plasma membranes as free cholesterol [34,35,36]. *Acat2* has long been suggested to play a key role in facilitating cholesterol absorption by esterifying free cholesterol and directing it to chylomicron assembly and secretion. In fact, studies in *Acat2* knockout mice demonstrate that the loss of cholesterol ester formation inhibits cholesterol absorption [37,38]. Our findings suggest that lowering PS content in infant formula, in addition to cholesterol supplementation, will increase dietary cholesterol uptake by the ileum.

Cholesterol synthesis was not directly measured in the present study. Instead, two intermediates of the cholesterol pathway (desmosterol and lathosterol) were determined as proxies for synthesis rates. Circulating cholesterol precursor markers have been used in other studies examining cholesterol synthesis in vivo [39]. As indicated by plasma D:C and L:C ratios, cholesterol synthesis was suppressed primarily in piglets fed the FC-LP diet, containing the low PS and high cholesterol concentrations. Corresponding to this apparent decrease in cholesterol synthesis, nuclear SREBP2 expression was also reduced in both F-LP and FC-LP groups, which underscores the importance of PS removal from the diet in reducing cholesterol synthesis rates. However, it should be noted that the cholesterol precursor ratios are only proxies for cholesterol synthesis. Direct measures of synthesis, i.e., fractional synthesis rates (FSR), would have been an asset in this study.

Our results reveal that the content of both PS and cholesterol in infant formulas may affect cholesterol metabolism and synthesis. These findings are consistent with other published studies. For example, Bayley et al. investigated the effects of supplementing formula with cholesterol on de novo cholesterol synthesis in formula fed infants compared with breastfed infants, and demonstrated higher fractional cholesterol synthesis rates in formula fed infants despite supplementation with cholesterol [10]. In our study, the supplementation of cholesterol to a regular infant formula resulted in reduced cholesterol synthesis, but to a lesser extent than the combination with the reduced PS content, as revealed by some cholesterol synthesis markers. This therefore highlights the potential value of reducing PS in infant formula to achieve a favorable cholesterol synthesis profile. This could potentially have long-term effects. A recently published study showed the consequences of decreased milk cholesterol availability, early in life, on the metabolism of cholesterol in adulthood in a mouse model [40]. The authors blocked the intestinal absorption of cholesterol in milk fed to newborn mice by supplementing the food of dams with ezetimibe, which interferes with the intestinal cholesterol absorption, and revealed a decrease in cholesterol absorption and an increased histone methylation in small intestine tissues from 24-week old offspring.

## 5. Conclusions

Our findings point to the importance of sterol profiles in infant nutrition for short-term cholesterol balance. The study demonstrated that infant formula should not only provide the cholesterol, but also enable its absorption, in order to ensure optimal cholesterol metabolism. These findings speculate to impact the long-term effect of dietary cholesterol on health well beyond infancy. 

## Figures and Tables

**Table 1:** Diet composition of the four experimental infant formulas: F-HP, F-LP, FC-HP, and FC-LP.

	Formula			
	F-HP	F-LP	FC-HP	FC-LP
Energy, kCal	513	512	514	512
Protein, g	12	12	11.9	11.9
Fat, g	26.6	26.5	26.7	26.5
Carbohydrate, g	56.5	56.3	56.4	56.4
**Sterols**				
Cholesterol, mg	22.5	24.2	85.6	84.2
PS, mg	79.2	9.5	79.1	9.9
**Minerals**				
Calcium, mg	381	395	375	380
Phosphorus, mg	284	302	296	297
Potassium, mg	491	515	489	488
Sodium, mg	147	152	152	144
Chloride, mg	360	360	360	360
Iron, mg	5.87	6.31	5.76	5.7
Magnesium, mg	43.7	46.1	44.8	46
Manganese, µg	316	321	309	310
Zinc, mg	5.49	5.54	5.45	5.55
Copper, µg	438	399	412	439
Iodine, µg	130	126	124	116
Selenium, µg	25.4	26	26.2	23.4
**Vitamins**				
Vitamin A, µg	699.4	708	743	685.5
Vitamin D, µg	8.1	8.8	8.3	8.4
Vitamin E, mg	12.4	17.2	13	16.5
Vitamin K, µg	38	33.4	39	41.3
Thiamine, µg	790	790	790	810
Vitamin B2, µg	1300	1300	1300	1300
Pyridoxine, µg	650	690	680	660
Vitamin B12, µg	1.9	1.9	1.9	1.9
Niacin, µg	5000	5000	5000	5000
Folic acid, µg	121	130	126	129
Pantothenic acid, µg	4800	4800	4000	4400
Biotin, µg	27.5	27.5	28.1	28.4
Vitamin C, mg	89.4	89.4	84.2	25.2
Choline, mg	145.6	147.6	144.9	143.1
Taurine, mg	49	49	49	50

Nutrient components are expressed as grams (g), milligrams (mg), or micrograms (µg) per 100 g of air dried powder. Each formula was produced using skim milk powder (147 g/kg), demineralized whey powder (300 mg/kg), whey protein concentrate 80% (37.3 g/kg), lactose (232 g/kg), and vegetable oil blend (263 g/kg). F-HP, formula containing high PS concentrations; F-LP, formula containing low PS concentrations; FC-HP, formula containing high cholesterol and PS concentrations; FC-LP, formula containing low cholesterol and PS concentrations.

**Table 2:** Mean daily dry matter intake and daily body weight gain for the piglets receiving the test infant formulas.

Formula	F-HP	F-LP	FC-HP	FC-LP	Overall	Overall	Overall
					SE	*p*-Value	Significance
Daily dry matter consumption (g)							
Week 1	129	133	129	133	8.2	0.962	NS
Week 2	155	162	152	161	9.6	0.864	NS
Week 3	191	201	188	198	11.8	0.857	NS
% consumed	97	98	96	98	0.8	0.229	NS
Daily body weight gain (g)							
Week 1	85	96	78	91	5.3	0.141	NS
Week 2	120	128	118	122	8.2	0.839	NS
Week 3	145	150	128	149	9.2	0.371	NS
Weeks 1–3	117	124	108	121	7.2	0.439	NS

Data are means, *n* = 8/diet. The % diet consumed was calculated as the amount of infant formula dry matter consumed divided by the amount of infant formula dry matter given to each piglet during the three-week feeding period. Statistical significance was determined by One-way ANOVA, followed by Tukey post hoc analysis. SE, overall standard error of the mean; NS, no overall significance, *p* ≥ 0.05. F-HP, formula containing high PS concentrations; F-LP, formula containing low PS concentrations; FC-HP, formula containing high cholesterol and PS concentrations; FC-LP, formula containing low cholesterol and PS concentrations.

**Table 3:** Apparent ileal digestibility and ileal digestibility content.

Formula	F-HP	F-LP	FC-HP	FC-LP	Overall	Overall	Overall
					SE	*p*-Value	Significance
Apparent ileal digestibility (% from formula)	31.8	66.3	73.0	85.0	4.87	<0.001	S
Apparent ileal digestibility content (g/kg formula)	69.8	160.4	625.0	710.0	26.2	<0.001	S

Data are means, *n* = 8/diet. Apparent ileal digestibility and digestibility content were determined as described in the Materials and Methods section. Statistical significance was determined by one-way ANOVA, followed by Tukey post hoc analysis. SE, overall standard error of the mean; S, significant, *p* ≤ 0.05. F-HP, formula containing high PS concentrations; F-LP, formula containing low PS concentrations; FC-HP, formula containing high cholesterol and PS concentrations; FC-LP, formula containing low cholesterol and PS concentrations.

**Table 4:** Cholesterol precursor concentrations in plasma and liver samples of piglets fed formulas with varying PS and cholesterol content.

Formula	Plasma		Liver	
	D:C ratio	L:C ratio	D:C ratio	L:C ratio
F-HP	0.25 ± 0.03 ^a^	0.14 ± 0.02 ^a^	0.42 ± 0.03 ^a^	0.42 ± 0.03 ^a^
F-LP	0.19 ± 0.02 ^a^	0.12 ± 0.01 ^a^	0.18 ± 0.01 ^b^	0.37 ± 0.04 ^a,b^
FC-HP	0.22 ± 0.02 ^a^	0.10 ± 0.01 ^a^	0.37 ± 0.02 ^a^	0.24 ± 0.02 ^b^
FC-LP	0.14 ± 0.01 ^b^	0.07 ± 0.01 ^b^	0.19 ± 0.01 ^b^	0.20 ± 0.01 ^b,c^
*p* Values				
PS	0.010	0.199	<0.001	0.057
Cholesterol	0.106	0.031	0.568	<0.001
Interaction	0.682	0.241	0.434	0.499

Data are means ± SEM for piglets fed infant formulas containing either high PS and low cholesterol concentrations (F-HP), low PS and low cholesterol concentrations (F-LP), high PS and high cholesterol concentrations (FC-HP), and low PS and high cholesterol concentrations (FC-LP); *n* = 8/diet. Abbreviations: desmosterol-to-cholesterol ratio (D:C ratio), lathosterol-to-cholesterol ratio (L:C ratio). Significant differences were determined by two-way ANOVA, followed by Student Newman-Keuls post hoc analysis; labeled means in a column without a common letter differ, *p* < 0.05.

## Supplementary Materials

The following are available online at http://www.mdpi.com/2072-6643/10/12/1848/s1, Table S1: Plant sterol concentrations in plasma and liver samples from piglets fed infant formulas containing different PS and cholesterol concentrations.
