# Supplementary material for: Modulating Sterol Concentrations in Infant Formula Influences Cholesterol Absorption and Synthesis in the Neonatal Piglet
Source: Nutrients. 2018 Dec 1;10(12):1848. doi: 10.3390/nu10121848 (PMC6316586; doi:10.3390/nu10121848)
Supplement: Supplementary file 1 [file nutrients-10-01848-s001.pdf]

Supplementary Table S1. Plant sterol concentrations in plasma and liver samples from piglets fed infant formulas containing different PS and cholesterol concentrations.

	F-HP	F-LP	FC-HP	FC-LP	*P* values		
					PS	Cholesterol	Interaction
			Plasma				
Brassicasterol, mg/100mls	<LOD	<LOD	<LOD	<LOD	--	--	--
Campesterol, mg/100mls	2.72 ± 0.67^{a}	0.44 ± 0.07^{b}	3.74 ± 0.38^{a}	0.55 ± 0.03^{b}	<0.001	0.146	0.267
Sitosterol, mg/100mls	1.22 ± 0.23^{a}	0.34 ± 0.04^{b}	1.09 ± 0.17^{a}	0.32 ± 0.02^{b}	<0.001	0.608	0.728
Sitostanol, mg/100mls	0.13 ± 0.01^{a}	0.11 ± 0.01^{a}	0.15 ± 0.02^{a}	0.12 ± 0.02^{a}	0.091	0.428	0.844
			Liver				
Brassicasterol, mg/100g	<LOD	<LOD	<LOD	<LOD	--	--	--
Campesterol, mg/100g	6.25 ± 0.66$_{a}$	0.94 ± 0.09^{b}	6.02 ± 0.65^{a}	0.88 ± 0.04^{b}	0.091	0.852	0.755
Sitosterol, mg/100g	1.78 ± 0.20^{a}	0.44 ± 0.04^{b}	1.32 ± 0.17^{a}	0.41 ± 0.02^{b}	<0.001	0.084	0.140
Sitostanol, mg/100g	0.37 ± 0.06^{a}	0.28 ± 0.04^{a}	0.28 ± 0.05^{a}	0.172 ± 0.01^{a,b}	0.044	0.038	0.788

PS concentrations were determined by GC-MS as previously described (17); n=8/diet. Data are means ± SE for piglets fed infant formulas containing either high PS and low cholesterol concentrations (F-HP), low PS and low cholesterol concentrations (F-LP), high PS and high cholesterol concentrations (FC-HP), and low PS and high cholesterol concentrations (FC-LP). Significant differences were determined by Two-way ANOVA followed by Student Newman-Keuls *post hoc* analysis; labeled means in a column without a common letter differ, $P < 0.05$.
